# EuGMS 2019 Congress report: evidence-based medicine in geriatrics

**DOI:** 10.1007/s41999-020-00416-w

**Published:** 2020-10-13

**Authors:** Suzy V. Hope, Anastasia Koutsouri, Sylvain Nguyen, Karolina Piotrowicz, Mirko Petrovic, Jerzy Gasowski

**Affiliations:** 1 College of Medicine and Health, University of Exeter, Exeter, UK; 2 Department of Healthcare for Older People, Royal Devon and Exeter NHS Foundation Trust, Exeter, UK; 3 Outpatient Geriatric Department, Henry Dunant Hospital Center, Athens, Greece; 4 Service of Geriatric Medicine and Geriatric Rehabilitation, University of Lausanne Hospital Center, Lausanne, Switzerland; 5 Department of Internal Medicine and Gerontology, Faculty of Medicine, Jagiellonian University, Kraków, Poland; 6 Section of Geriatrics, Department of Internal Medicine and Paediatrics, Faculty of Medicine and Health Sciences, Ghent University, Ghent, Belgium

**Keywords:** EuGMS, Geriatric medicine, Evidence-based medicine, Congress, Kraków

## Abstract

The 2019 EuGMS Congress “Evidence-Based Medicine in Geriatrics” was held in Krakow, Poland, and attended by over 1600 participants from 64 different countries. A summary and reflection on the congress was presented in the Closing Ceremony by European Academy for Medicine of Aging graduates, and summarised in this article. Keynote lectures, ‘state of the art’ sessions and symposia presented the evidence relating to different age-related conditions, their prevention, management and treatments. Hot topic areas included frailty and multimorbidity, and evidence-based attempts to address these conditions at different life stages. The field of geriatrics represents unique challenges for evidence-based medicine practice. There is much research going on. Clear leadership is needed to facilitate consensus agreements on standard definitions, methods and relevant outcomes, in collaboration with older people themselves, to maximise the opportunities and benefits of doing this research, and benefiting our patients and society at large.

## Key summary points

**Aim:**

To report on the 2019 EuGMS Congress in Krakow.

**Findings:**

Evidence-based medicine in geriatrics is a previously neglected, now rapidly expanding field. Heterogeneity of our older population brings many questions and challenges for research.

**Message:**

Personalized approaches based on evidence-based practices, standardisation of definitions and meaningful outcomes, in collaboration with older people themselves, and with other specialties, are the new frontiers and challenges for research.

## Introduction

Evidence-based medicine (EBM) was first described by David L. Sackett as the “conscientious, explicit and judicious use of the best current clinical research evidence in making decisions about the care of patients”[1]. This, however, may not be straightforward in geriatric medicine: heterogeneity can be difficult to take account of in classic randomized controlled trials (RCTs), with the widespread use of age or comorbidity cut-offs [2], in addition to practical recruitment issues. It can thus be challenging to answer with EBM the sorts of questions we and our older patients may have. To address this issue, the 15th EuGMS congress was dedicated to EBM. This brief report picks out some of the many topics covered.

## Evidence-based medicine in geriatrics

**Fig. 1:**
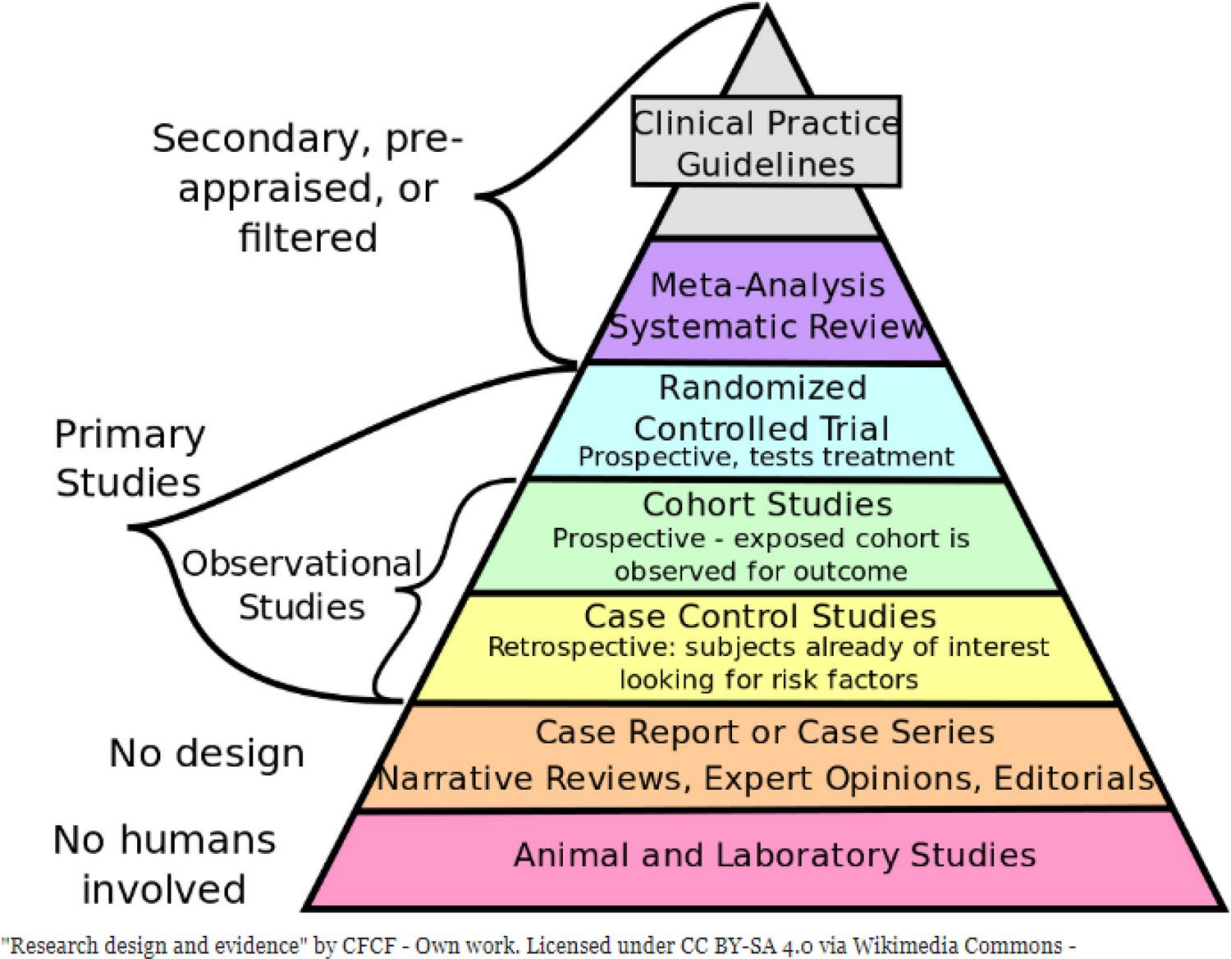
Evidence-based medicine [3]

**Fig. 2:**
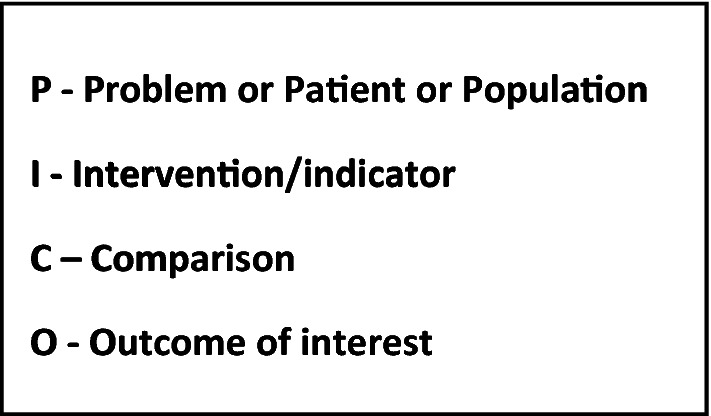
PICO model for clinical or research questions

The traditional pyramid of evidence-based medicine is evolving [3], Fig. 1. Traditionally, older patients have been systematically excluded from high-quality RCTs [2, 4] or those included are mainly the ‘robust’ ones, still fitting more ‘single disease’ criteria. Straightforward extrapolation of apparently robust evidence from studies in younger people and often single condition studies, is not often appropriate in more frail older patients with potentially several comorbidities. The complex interplay of physiological, psychological and social differences makes each older patient—and their potential response to a given treatment or situation—unique. Designing “traditional” randomised control trials are rarely possible in this group, because of the complexities involved in the research questions, the patients, and the healthcare systems. In studying multimorbidity, for example, trying to define which and how many diseases count, or even finding standard disease classifications can lead to real issues in designing practical, meaningful and reproducible study designs. Attempts at systematic reviews and meta-analyses are made, using the PICO question (Fig. 2). But the complexities previously mentioned mean that consensus statements, incorporating the myriad of different trials (with varying inclusion and exclusion criteria, designs and outcomes) with expert opinions, are often needed. None of this is easy, however. Franz Messerli in his opening lecture, described clearly the rift sometimes apparent between those who preach, teach and treat: part of this confusing scenario may result from the fact that those very few who preach rarely treat, those few more who teach only sometimes treat, and those many who treat do not always listen, neither to those who preach nor to those who teach!

## EBM in the prevention and healthy ageing

A resounding message from the congress was that exercise at every stage of life is vital and we should promote it much more. It should be viewed as a medicine—consider recommending a dose, a time and a frequency. Regular physical activity can reduce the risk of hip fracture by up to 68%, type 2 diabetes up to 40%, cardiovascular disease by up to 35%, dementia, depression, colon cancer and all-cause mortality each by up to 30% [5]. Multiple presentations showed the interest and ongoing research in the field of prehabilitation, particularly ahead of oncology treatment and surgical treatments. Increasing evidence suggests prehabilitation can result in significantly improved return to health, but that larger-scale RCTs (especially in oncology) are still needed.

A submitted symposium highlighted the increasing evidence of an interplay between gut microbiota and physical function [6], cognitive health and infections—but there is still much we do not understand about the gut and gut physiology in ageing.

Stefania Maggi told us in a Keynote session that vaccination annually saves > 2.5 million deaths related to communicable diseases. At the same time reduction in all cardiovascular events was associated with vaccination [7]. This should be considered as a new outcome in clinical trials for vaccines.

## EBM in age-related conditions

The results from the European MID-Frail study were presented, a 12-month multimodal intervention in older people with type 2 diabetes. This demonstrated improvements in frailty and quality of life, and in a cost-effective manner [8]. This is important as diabetes is a risk factor for premature disability and dementia, frailty is a major risk factor in increased risk of death or disability, and the rates of all are increasing.

Managing blood pressure in older people is still a challenge and with ongoing debate and current research looking at optimal target blood pressure measurements for our older and more frail population. Recalling the past can sometimes be illuminating, however: attendees were astonished at the high systolic blood pressures people lived with only a few decades ago [9].

The updated European guidance for the Diagnosis and Management of Osteoporosis in Postmenopausal Women were presented [10]. Surprising evidence showed the rates of osteoporosis prescriptions has fallen over the last two decades, despite the advent of orthogeriatrics as a subspeciality in many countries. We need to continue assessing risk and trying to ensure if it occurs, and adhere to the mantra the “first break is the last”.

A dedicated session on ageing with HIV highlighted the fact that geriatricians most probably will encounter HIV survivors due to the advances made in HIV treatment over the last decades.

Cancer frequency increase with age. At present most cancer treatment approaches remain tumour-centric, although frailty has been shown to predict complication rates. At present, the majority of oncology multidisciplinary team meetings (MDTs) do not consider frailty or “patient-related” factors. Early EBM in this field demonstrated incorporation of comprehensive geriatric assessment (CGA) into oncology MDTs led to adjusted management in > 70% of cases, reduced complications and reduced 90-day mortality (Ommundsen et al., unpublished).

## EBM in frailty and multimorbidity

A recent systematic review which looked at the effectiveness of interventions to prevent pre-frailty and frailty progression in older adults also summarised current evidence and research gaps [11]. As with CGA, multimodal interventions seem to be developing the greatest evidence base in many of these areas. This will need the incorporation of economic evaluations to future studies, to justify potentially multimodal (expensive!) interventions.

Amaia Calderon-Larrañaga and colleagues have shown distinctly different patterns of multimorbidity in difference people, with significantly different sociodemographic, lifestyle, clinical, and functional profiles [12]. This could potentially enable targeted preventive approaches.

A consensus report from the global clinical nutrition community presented the new Global Leadership Initiative on Malnutrition (GLIM) criteria for the diagnosis of malnutrition, involving a two-step approach: first screening to identify “at risk” status by the use of any validated screening tool, and second, assessment for diagnosis and grading the severity of malnutrition [13].

A recent Cochrane review on interventions to improve the appropriate use of polypharmacy for older people was inconclusive, largely due to the very limited number of studies without significant bias [14]. This reflects again the recurring issue of there being many different tools in use and little standardisation.

## Conclusions

Geriatrics represents unique challenges for evidence-based medicine practice. Finbarr Martin pulled all these observations and reflections together in the Presidential Keynote Lecture: ‘twenty-first-century geriatric medicine for twenty-first-century populations’.

Multimorbidity, frailty, resilience, disability are all complex concepts which take geriatric medicine beyond simple medicine. The person-centred approach in geriatrics is fundamental to ensuring we focus on the meaningful outcomes, and not be distracted by the ‘easily measurable’ alone. Luckily, there are many exciting and high-quality research and clinical improvement projects going on throughout Europe!

We would suggest that strong EuGMS leadership is needed to help facilitate consensus agreements on standard definitions, methods and relevant outcomes, in collaboration with patients and the public, other specialist societies (perhaps in particular the gerontological societies), to maximise the opportunities and benefits of doing meaningful research with the aim of working with and benefiting our patients and society at large. New technologies for example may help in empowering the older population further.

Since drafting this summary a consensus statement calling for alignment of common data measurements and outcomes has been published by numerous leading researchers in geriatrics, frailty and ageing [15].

In the meantime, the COVID-19 pandemic has affected the whole world and especially our patients. Moreover, major issues such as climate change are going to drastically affect the future, and should proactively be taken into consideration at all points. New frontiers in geriatrics have emerged and the need for strong leadership, collaboration and use of new technologies have become indispensable.
